# Dendritic Cells Loaded with Heat Shock-Conditioned Ovarian Epithelial Carcinoma Cell Lysates Elicit T Cell-Dependent Antitumor Immune Responses *In Vitro*

**DOI:** 10.1155/2019/9631515

**Published:** 2019-11-25

**Authors:** Iván Flores, Daniel Hevia, Andrés Tittarelli, Dafne Soto, Daniel Rojas-Sepúlveda, Cristián Pereda, Fabián Tempio, Camila Fuentes, Cristian Falcón-Beas, Jimena Gatica, Felipe Falcón-Beas, Mario Galindo, Flavio Salazar-Onfray, Fermín E. González, Mercedes N. López

**Affiliations:** ^1^Disciplinary Program of Immunology, Institute of Biomedical Sciences, Faculty of Medicine, Universidad de Chile, 8380453 Santiago, Chile; ^2^Millennium Institute on Immunology and Immunotherapy, Faculty of Medicine, Universidad de Chile, 8380453 Santiago, Chile; ^3^Laboratory of Cellular and Molecular Biology, Institute for Research in Dental Sciences, Faculty of Dentistry, Universidad de Chile, 8380492 Santiago, Chile; ^4^Institute of Biomedical Sciences, Faculty of Medicine, Universidad de Chile, 8380453 Santiago, Chile; ^5^Laboratory of Experimental Immunology & Cancer, Department of Conservative Dentistry, Faculty of Dentistry, Universidad de Chile, 8380492 Santiago, Chile

## Abstract

Ovarian epithelial carcinoma (OEC) is the most frequent ovarian tumor, characterized by a high mortality in advanced stages where conventional therapies are not effective. Based on the role of the immune system in the progression of this disease, immunotherapy using checkpoint blockade has been considered as a therapeutic alternative. Nevertheless, its results do not match up to the positive results in entities like melanoma and other malignancies, suggesting the need to find other therapies to be used alone or in combination. Dendritic cell- (DC-) based vaccines have shown promising results in several types of cancer, such as melanoma, prostate, and lung cancers, due to the essential role played by DCs in the activation of specific T cells, thus using other ways of activating the immune response than immune checkpoint blockade. During the last decade, we have used DC-based vaccines loaded with an allogeneic heat shock-conditioned melanoma cell lysate in the treatment of advanced stage patients in a series of clinical trials. In these studies, 60% of treated patients showed immunological responses which correlated positively with improved survival. Considering the relevance of ovarian cancer and the promising results of our DC-based vaccine, we show here that heat shock-conditioned cell lysates derived from ovarian epithelial carcinoma cell lines have the potential to induce the phenotypic and functional maturation of human DC, which in turn, is able to induce an efficient CD4^+^ and CD8^+^ T cell-mediated immune responses against ovarian cancer cell lines *in vitro*. In summary, OEC heat shock-conditioned cell lysate-loaded DCs may be considered for future combined immunotherapy approaches against ovarian tumors.

## 1. Introduction

Ovarian epithelial carcinoma (OEC) is a highly aggressive ovarian tumor that affects the female population with more than 150,000 deaths per year [1]. The OEC is the most frequent ovarian-associated tumor, representing about 85-90% of the ovarian tumor diagnoses [2] with a mortality of 4.5 deaths per 100,000 inhabitants. The overall survival at 5 years is 45%, decreasing to 27% in advanced stages of OEC [3, 4]. Usually, OEC is diagnosed based on symptoms such as abdominopelvic pain and abdominal distension, which are linked to other pathologies, making OEC accurate diagnosis very difficult [5].

The participation of the immune system and its adequate antitumor activity is crucial in the progression of the disease. It has been demonstrated that the infiltration of effector T cells into the tumor site is associated with a better prognosis and prolonged survival [6]. On the other hand, the presence of Treg cells in the tumor site and in ascites of OEC patients correlates with a poor prognosis [7], and patients who have low CD8^+^ T cell infiltration increase their probability of dying by OEC [8].

The evasion from the immune response is critical for tumor progression, and it is based, among other things, in the recruitment of proangiogenic immunosuppressive dendritic cells (DC), with low antigen presentation properties [9, 10], usually called tolerogenic/regulatory/dysfunctional DCs [11, 12]. In addition, OEC evades the immune response through the expression of the PD-1/PD-L1 complex, immunological inhibitory checkpoint molecules, necessary to maintain immune tolerance [13].

Today, the most promising immunotherapies against cancer are based on immune checkpoint blockers (ICB) against CTLA-4 or PD-1/PD-L1. These immunotherapies have shown to induce effective clinical responses in patients with melanoma, lung cancer, and other solid tumors [14–21]. Despite their relevant clinical outcomes, only 15% of OEC patients are responder to ICB treatment [22], strongly suggesting the need for complementary treatments, for example, active immunizations, which could improve the response rates of ICB. In this context, an alternative to increase the response rate of ICB is DC-based immunotherapies, which can be used as complementary treatments in cancer patients, with the advantage of enhancing an antitumor immune response via the activation of specific CD4^+^ and CD8^+^ T cells [23]. It has been reported that in OEC, autologous DCs loaded with ovarian-associated antigens (ErbB2, MUC-1, and CA-125) were able to stimulate the proliferation of autologous T cells and to induce tumor-specific cytotoxic activity [24, 25]. Additionally, the use of autologous lysates as a source of antigens for DC vaccines has shown promising results as a potential therapy against OEC. Furthermore, the combination of this treatment with antiangiogenic monoclonal antibodies to increase the clinical efficiency of the therapy, induced a 2-year prolonged survival of treated patients [26, 27]. In summary, DC-based immunotherapy could help to break the tolerance of the ovarian tumor microenvironment through the modulation of specific T cell response against OEC and synergize with ICB, enhancing patients' response.

Over the past decade, our laboratory has developed an autologous DC-based immunotherapy directed against advanced malignant melanoma, called TAPCells (tumor antigen-presenting cells), which consist in *ex vivo*-generated monocyte-derived DC, loaded with an allogeneic heat shocked melanoma lysate that is able to activate the immune system and induce a specific antitumor response in cancer patients [28]. Approximately 60% of treated patients respond to the treatment, showing a posttreatment survival time threefold higher (33 months) than that in nonresponder patients (11 months) [28]. The use of allogeneic heat shock-conditioned tumor cell lysates provides a vast number of different tumor-associated antigens described for melanoma and also delivers different damage-associated molecular patterns (DAMPs) induced by the heat shock, such as high mobility group box-1 (HMGB1) and plasma membrane translocated calreticulin (CRT) necessary for the proper maturation, activation, and cross-presentation of tumor-associated antigens by DCs, enhancing their antitumor-induced responses [28–30]. Recently, we have extended our results to other types of tumors, such as prostate cancer and gallbladder cancer, using specific allogeneic heat shock-conditioned cell lysates for each tumor [31, 32].

The purpose of this study was to investigate the immunogenicity of several combinations of heat shock-conditioned tumor lysates derived from different OEC cell lines (OECCL) and their effect on the differentiation, maturation, and activation of DC, as well as their ability to induce T cell-mediated responses anti-OECCL *in vitro*. Our results suggest that human DCs loaded with specific combinations of heat shock-conditioned lysates from OECCL were able to induce T cell activation and effector responses against OEC tumor cells. These results allow us to characterize and optimize effective tumor lysates to improve DC-based therapeutic strategies against OEC.

## 2. Materials and Methods

### 2.1. Cell Lines and Heat Shock-Conditioned OEC Lysates

The OEC cell lines Hey, SKOV-3, A7280, and CAOV3 were gently donated by Garett Owen from the Pontificia Universidad Católica de Chile. The melanoma cell lines Mel1, Mel2, and Mel3 were cultured as described before [28, 29]. The OECCL were cultured in DMEM culture medium (Corning, USA) supplemented with 10% of fetal bovine serum (FBS) and 1% of broad-spectrum antibiotic cocktail (penicillin 100 U/L and streptomycin 100 *μ*g/mL), until ~85% of confluence. Cells were maintained at 37°C under 5% CO_2_ and 95% relative humidity. The cells were harvested using PBS/EDTA 0.05% (Corning, USA), washed with PBS buffer, and counted using Neubauer's chamber.

For mixed OEC lysate (called MOVL) production, cells were mixed in equal amounts to achieve a final concentration of 4 × 10^6^ cells/mL in AIM-V culture medium (Thermo Fisher Scientific, USA). The mixed cell lysates evaluated were made as follows: MOVL1 (CAOV3+SKOV3+Hey) and MOVL2 (CAOV3+SKOV3+A2780). The mixed cells were subjected to heat shock stress by culturing them to 42°C for 1 hour plus 37°C for two additional hours, as previously described [28, 29]. Then, the cells were lysed by three freeze-thawing cycles (liquid nitrogen and then at 37°C, respectively). A heat shock-conditioned lysate from Hey cells was elaborated from 4 × 10^6^ cells/mL as described for the mixed cell lysates. The melanoma heat shock-conditioned lysate TRIMEL was produced as described before [28, 29]. Protein quantification of the lysates was made by a micro-Bradford colorimetric method.

### 2.2. Antibodies

Monoclonal antibodies (mAbs) against human carcinoembryonic antigen (CEA; clone COL-1), erbB2 (clone 3B5), and survivin (clone 8E2) were purchased from Thermo Fisher Scientific (Waltham, Massachusetts, USA). mAbs against human mucin-1 (MUC1; clone HMFG1), cancer antigen 125 (CA-125; clone SPM110), and calreticulin (clone FMC 75) were purchased from Abcam (Cambridge, USA). mAbs against human CD3-eFluor450 (clone SK7), human leukocyte antigen- (HLA-) DR, HLA-DQ, HLA-DP-PE (clone TU39), HLA-A, HLA-B, HLA-C-PE (clones W6/32), CD83-PECy7 (clone HB15e), CD11c-APC (clone 3.9), CD86-FITC (clone 2331), CD80-FITC (clone 2D10.4), interleukin- (IL-) 4-APC (clone 8D4-8), and IFN-*γ*-FITC (clone 4S.B3) were purchased from eBioscience (San Diego, CA, USA). mAbs against human CD25-FITC (clone 2A3), C-C chemokine receptor type 7- (CCR7-) PE (clone G043H7), C-X-C motif chemokine receptor (CXCR) 3-APC (clone 1C6/CXCR3), granzyme-B-PE (clone GB11), perforin-PE (clone *δ*G9), and IL-17A-PE (clone SCPL1362) were purchased from BD Biosciences (Franklin Lakes, NJ, USA). mAbs against human CD40-FITC (clone HI40a), CD4-PerCP Cy5.5 (clone MEM241), CD8-APC-h7 (clone MEM31), and CD4-FITC (clone MEM241) were purchased from Thermo Fisher (Waltham, MA, USA). As isotype control, IgG1 antibodies conjugated to FITC, PE, PerCP Cy5.5, or APC were used (eBioscience; San Diego, CA, USA).

### 2.3. Flow Cytometry

The surface expression of different molecules in OECCL, DCs, and T cells was analyzed by flow cytometry. Intracellular staining was performed with the Foxp3/Transcription Factor Fixation/Permeabilization Concentrate and Diluent kit (eBioscience). A live/dead kit (Thermo Fisher) was used for live/dead cell discrimination. Flow cytometry was conducted on a FACSVerse flow cytometer (BD Biosciences), and data analysis was performed using the FlowJo software (Tree Star, Inc., Ashland, OR, USA). For CRT translocation determination, OEC cells were processed for flow cytometry analysis after heat shock treatment (one hour at 42°C plus two additional hours at 37°C).

### 2.4. HMGB1 ELISA

Supernatants from OEC cells were collected after heat shock treatment (one hour at 42°C plus two additional hours at 37°C). The concentration of HMGB1 in 100 *μ*L of supernatants from the control and heat shocked OECCL (4 × 10^6^ cells/mL) was measured by ELISA using a specific HMGB1 ELISA kit according to the manufacturer's instructions (Cloud-Clone Corp.). 450 nm optical densities were measured in a Sunrise absorbance reader (Tecan).

### 2.5. DC Generation

Plastic adherent monocytes isolated from peripheral blood mononuclear cells (PBMC) of healthy donors from the Centro Metropolitano de Sangre y Tejidos, Hospital Metropolitano (Santiago, Chile), were cultured in serum-free AIM-V medium (Invitrogen) for 22 hours with 500 U/mL recombinant human IL-4 (rhIL-4; US-Biological) and 800 U/mL recombinant human granulocyte-macrophage colony-stimulating factor (rhGM-CSF; Sheering Plough) and then stimulated for 24 hours with 100 *μ*g/mL of OECCL heat shock-conditioned lysates (Hey, MOVL1, or MOVL2), heat shock-conditioned melanoma cell lysate (TRIMEL), and LPS (1 *μ*g/mL) or with medium alone (activated monocytes (AM)) as previously described [28, 29].

### 2.6. DC/T Cell Cocultures

For allogeneic cell cocultures, peripheral blood lymphocytes (PBL) were obtained from healthy donors and cocultured for 5 days with TRIMEL-DCs, DCs matured with OECCL lysates (MOVL1-DCs or MOVL2-DCs), AM, or LPS-DCs at ratios DC : PBL of 1 : 10 and 5 : 1, in RPMI 1640 medium supplemented with 10% FBS and 150 IU/mL rhIL-2 (Proleukin). For autologous cell cocultures, sorted CD4^+^ and CD8^+^ T cells isolated from PBL of healthy donors were cocultured with AM, TRIMEL-DCs, Hey-DCs, MOVL1-DCs, or MOVL2-DCs for 14 days at a DC : T cell 1 : 10 ratio in RPMI 1640 medium supplemented with 10% FBS and 150 IU/mL rhIL-2. T cells were restimulated at day 7 with freshly prepared DCs maintaining the initial DC : T cell ratio. The surface expression of CD25 and CXCR3 was analyzed in CD4^+^ and CD8^+^ T cells by flow cytometry. For intracellular IFN-*γ*, IL-17A, and IL-4 staining, 1 × 10^6^ T cells were cultured for 4 hours at 37°C in RPMI 1640 medium with 10% FBS containing 1 *μ*g/mL ionomycin, 0.15 *μ*M phorbol myristate acetate (PMA), and 3 *μ*g/mL brefeldin-A.

### 2.7. IFN-*γ* ELISPOT

Sorted CD4^+^ T cells activated or not with autologous AM, TRIMEL-DCs, Hey-DCs, MOVL1-DCs, or MOVL2-DCs were cocultured with 1 × 10^4^ target cells: OECCL (Hey and CAOV3), melanoma cell line (Mel1), or K562 cells for 16 hours at different effector/target ratios. IFN-*γ* release was tested by an ELISPOT assay according to the manufacturer's instructions (ELISPOT Ready-SET-Go, eBioscience) as previously described [33].

### 2.8. Cytotoxicity Assay

The cytotoxic activity of CD8^+^ T cells against 1 × 10^4^ target cells: OECCL (Hey and CAOV3), melanoma cell line (Mel1), or K562 cells was measured by conventional 4-hour ^51^Cr release assays at different effector/target ratios as described previously.

### 2.9. Statistical Analysis

All values were expressed as the mean ± standard deviation (SD). Differences in means between two groups were analyzed by 2-tailed Student's *t*-test or, when data was not normally distributed, with a nonparametric Mann-Whitney *U* test. Comparison between multiple groups was analyzed using one-way ANOVA. When ANOVA showed significant differences, pairwise comparison between means was tested by Student's *t*-test or Mann-Whitney analysis. *p* values ≤ 0.05 were considered statistically significant. Analyses were performed using GraphPad Prism 6 software.

## 3. Results

### 3.1. OECCL Express a Wide Range of Ovarian Epithelial Cancer-Associated Antigens

**Figure 1:**
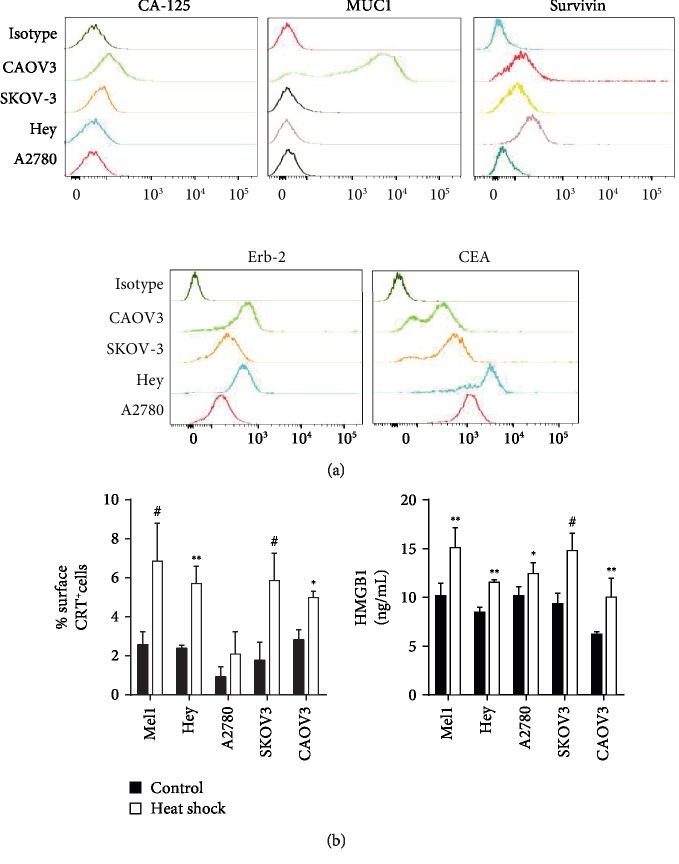
OECCL express OEC-associated antigens and increase DAMP production under heat shock treatment. (a) Representative histograms for CA-125, MUC1, survivin, ErbB-2, and CEA expression in four OECCL (CAOV3, SKOV-3, Hey, and A2780) evaluated by flow cytometry. The upper histograms indicate isotype control staining. (b) The levels of the plasma membrane translocated calreticulin (surface CRT, left panel) and the HMGB1 in the supernatant (right panel) of heat shock-treated (white bars) or control (black bars) OEC cells. The results were obtained from multiple independent experiments. ^∗^*p* < 0.05, ^∗∗^*p* < 0.01, and ^#^*p* < 0.001.

To select OECCL suitable for the production of cell lysates as the source of multiple tumor antigens, we first determined the expression levels of well-established OEC-associated antigens (CA-125, MUC1, ErbB-2, CEA, and survivin) [34, 35] in the CAOV3, SKOV-3, Hey, and A2780 cell lines by flow cytometry. We observed that all the OEC cell lines evaluated expressed ErbB-2 and CEA antigens (Figure 1(a)). The antigen CA-125 was expressed only by CAOV3 and in a lesser quantity by SKOV-3 cells. Only CAOV3 cells expressed the MUC1 antigen, and survivin was expressed by all the cell lines but not by A2780 cells (Figure 1(a)). Also, we observed differential abundance patterns of these antigens among OECCL: CAOV3 cells showed the higher expression level of CA-125 and MUC1, compared to the rest of OECCL, whereas CAOV3 and Hey cells showed the greater abundance of ErbB-2 expression. The expression level of CEA was higher in Hey cells. Given that CAOV3 and Hey cells showed the broader and higher expression pattern of OEC-associated antigens, we suggest that these cell lines must be included as part of OECCL mixture lysates destined as an ovarian tumor-associated antigen source for DC-based immunotherapy.

### 3.2. Heat Shock Treatment Induces DAMPs in OECCL

For almost 15 years, we have developed a DC-based vaccine that improves the long-term survival of patients with advanced melanoma [28, 30, 33]. This DC vaccine is manufactured using an allogeneic melanoma cell lysate composed of three human melanoma cell lines (named TRIMEL) as the source of melanoma-associated antigens. Moreover, previous to the lysate generation, the melanoma cell lines were conditioned with a 42°C heat shock protocol, in order to induce DAMPs such as the plasma membrane translocation of calreticulin (CRT) and the release of HMGB1 protein. We previously showed that these DAMPs act as activators of the DC vaccines [29]. Heat shock-induced plasma membrane translocated CRT and released HMGB1 mediated an optimal antigen-presenting cell (APC) maturation and antigen cross-presentation, providing a unique strategy to obtain efficient tumor antigen-presenting cells with a mature DC-like phenotype [29]. Here, we evaluated if the OECCL were able to induce those DAMPs in response to heat shock treatment. As showed in Figure 1(b), all the OECCL increased the level of surface CRT and release of HMGB1 after the heat shock treatment, with the only exception of the A2780 cell line that did not show significant changes in CRT exposure. As a positive control, we used the melanoma cell line Mel1 used in the elaboration of TRIMEL [29].

### 3.3. Heat Shock-Conditioned OEC Lysates Induce the Differentiation of Activated Monocytes into Mature DCs

**Figure 2:**
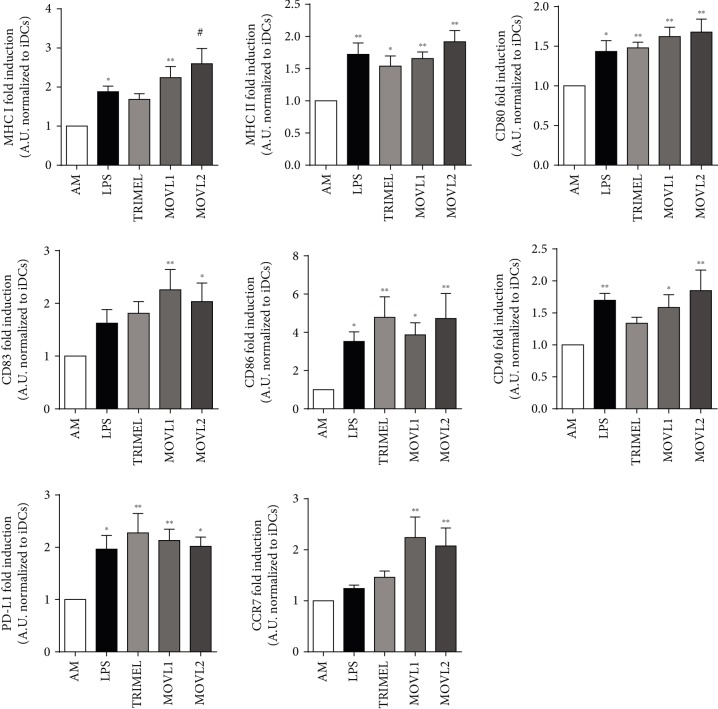
Heat shock-conditioned OECCL lysates induce maturation and activation-associated marker expression on monocyte-derived DCs. The surface expression of MHC I, MHC II, CD80, CD83, CD86, CD40, and CCR7 was evaluated by flow cytometry on CD11c^+^ IL-4/GM-CSF-activated monocytes unstimulated (AM) or stimulated for 24 hours with LPS (1 *μ*g/mL) and the heat shock-conditioned lysates TRIMEL, MOVL1, or MOVL2 (100 *μ*g/mL). The results were obtained from multiple independent experiments and are showed as fold change relative to unstimulated AM. Statistics compare treatments to AM; ^∗^*p* < 0.05, ^∗∗^*p* < 0.01, and ^#^*p* < 0.001.

We have previously reported that the addition of the heat shock-conditioned melanoma lysate TRIMEL to IL-4/GM-CSF-activated monocytes (AM) mediates the induction of canonical surface markers associated with DC maturation such as MHC I, MHC II, CD80, CD83, and CD86 [29]. To explore if heat shocked OECCL-conditioned lysates may induce similar results, we prepared two different OEC mixture lysates (MOVL) that included CAOV3+SKOV3+Hey (MOVL1) and CAOV3+SKOV3+A2780 (MOVL2). As observed in Figure 2, both MOVL1 and MOVL2 lysates induced the expression of maturation and activation markers (MHC I, MHC II, CD80, CD83, CD86, and CD40) on DCs. The levels of induction of these markers were similar to the ones observed in the positive controls: AM stimulated with LPS or with the TRIMEL lysate. In addition, the expression of the chemokine receptor CCR7, involved in the migration of DCs from periphery to lymph nodes [36], was strongly induced in AM incubated with MOVL1 and MOVL2 treatments. Altogether, these results indicate that the heat shocked OEC lysates induce the differentiation and activation of monocyte-derived DCs.

### 3.4. DCs Loaded with Heat Shock-Conditioned OEC Lysates Induce the Activation of Allogeneic CD4^+^ and CD8^+^ T Cells

**Figure 3:**
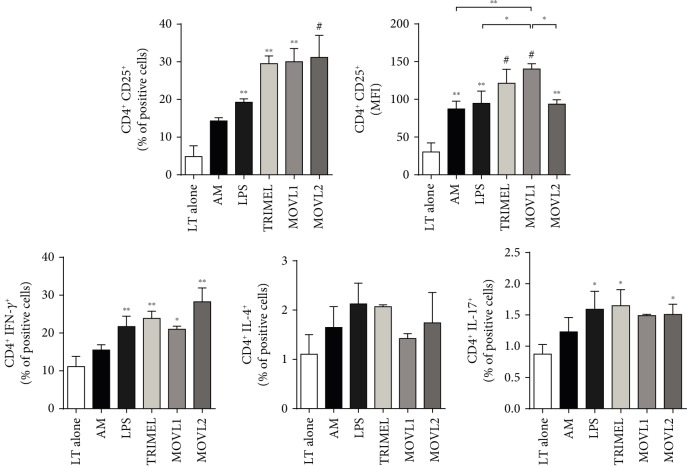
Activation of allogeneic CD4^+^ T cells by monocyte-derived DCs maturated with heat shock-conditioned OEC lysates. Peripheral blood lymphocytes (PBL) were kept alone (LT alone) or cocultured for 5 days with allogeneic IL-4/GM-CSF-activated monocytes unstimulated (AM) or stimulated for 24 hours with 1 *μ*g/mL LPS (LPS-DC) or 100 *μ*g/mL of heat shock-conditioned lysates TRIMEL (TRIMEL-DC) and MOVL1 or MOVL2 (MOVL1-DC and MOVL2-DC, respectively). The expression of CD25, IFN-*γ*, IL-4, and IL-17 was evaluated in the CD4^+^ T cells by flow cytometry. The results were obtained from multiple independent experiments. Statistics compare treatments to LT alone; ^∗^*p* < 0.05, ^∗∗^*p* < 0.01, and ^#^*p* < 0.001. Also, a comparison between MLOV1, LPS, and AM was made using the same *p* values.

**Figure 4:**
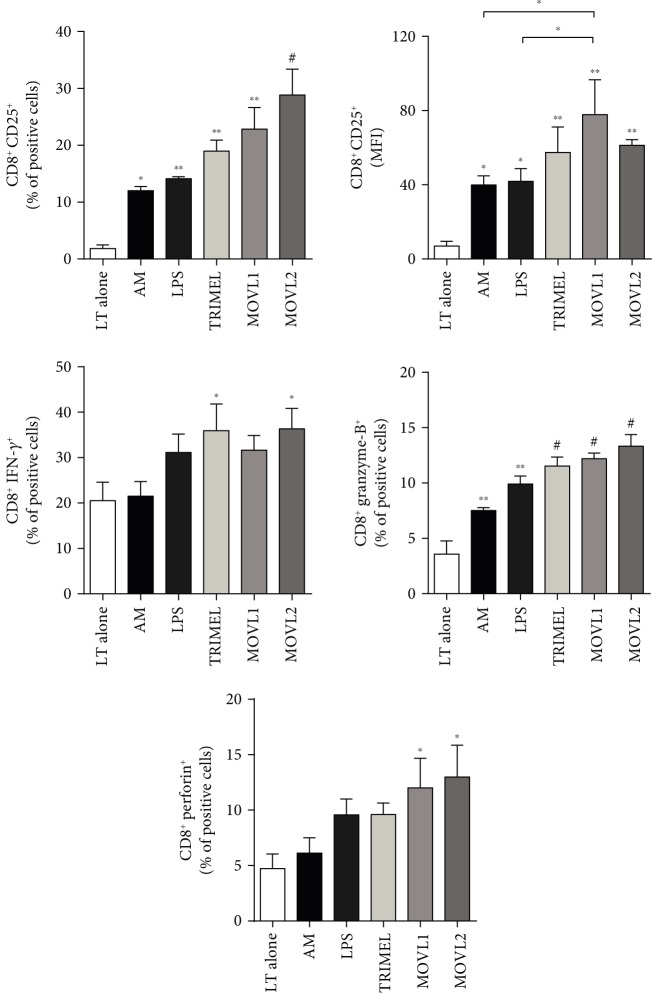
Activation of allogeneic CD8^+^ T cells by monocyte-derived DCs maturated with heat shock-conditioned OEC lysates. Peripheral blood lymphocytes (PBL) were kept alone (LT alone) or cocultured for 5 days with allogeneic IL-4/GM-CSF-activated monocytes unstimulated (AM) or stimulated for 24 hours with 1 *μ*g/mL LPS (LPS-DC) or 100 *μ*g/mL of heat shock-conditioned lysates TRIMEL (TRIMEL-DC) and MOVL1 or MOVL2 (MOVL1-DC and MOVL2-DC, respectively). The expression of CD25, IFN-*γ*, granzyme-B, and perforin was evaluated in the CD8^+^ T cells by flow cytometry. The results were obtained from multiple independent experiments. Statistics compare treatments to LT alone; ^∗^*p* < 0.05, ^∗∗^*p* < 0.01, and ^#^*p* < 0.001. Also, a comparison between MLOV1, LPS, and AM was made, using the same *p* values.

To determine if MOVL1 or MOVL2 lysates have the potential to induce functionally mature DCs, first, we investigated the capacity of DCs stimulated with these lysates to activate allogeneic T cells. We evaluated the surface expression of the T cell activation marker CD25, the effector profile cytokines IFN-*γ*, IL-4, or IL-17 on CD4^+^ T cells (Figure 3), and CD25, IFN-*γ*, granzyme-B, and perforin expression on CD8^+^ T cells (Figure 4) cocultured for 5 days with MOVL1- or MOVL2-DCs. All the DCs tested, including DCs stimulated with LPS (LPS-DC) or TRIMEL (TRIMEL-DC) (positive controls), induced the expression of CD25 on both T cell subsets (Figures 3 and 4). Our results also showed that CD4^+^ T cells cocultured with allogeneic DCs loaded with MOVL1 or MOVL2 lysates expressed higher levels of the Th1 cytokine IFN-*γ* and, in a lower extent, the Th17 cytokine IL-17 (Figure 3) than T cells cocultured with AM or kept alone, whereas the expression of IL-4 on CD4^+^ T cells (related to the Th2 profile) did not show any significant changes among different treatments. CD8^+^ T cells cocultured with allogeneic MOVL1- or MOVL2-DCs expressed higher levels of IFN-*γ* and of the effector molecules granzyme-B and perforin than T cells alone or stimulated with AM (Figure 4). Taken together, these results suggest that DCs stimulated with heat shock-conditioned OECCL lysates have the potential to activate allogeneic CD4^+^ and CD8^+^ T cells.

### 3.5. DCs Loaded with Heat Shock-Conditioned OEC Lysates Induce Effector Responses in Autologous CD4^+^ and CD8^+^ T Cells

**Figure 5:**
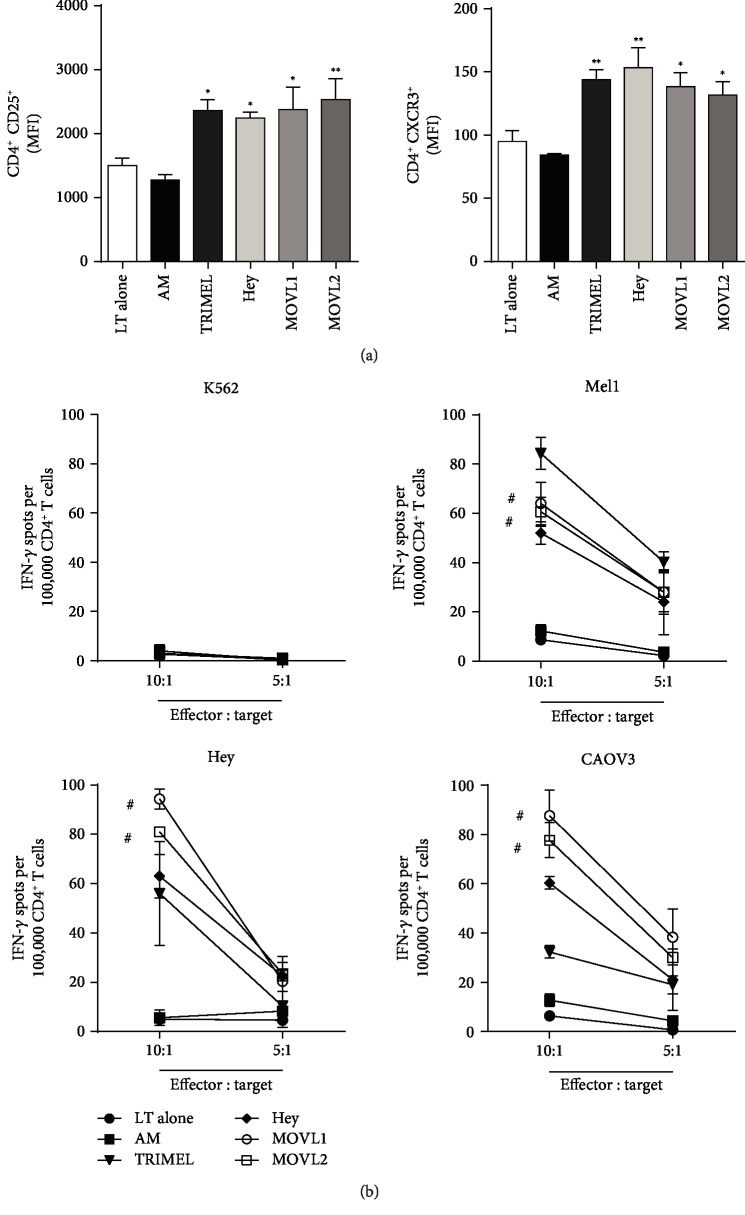
Activation of autologous T cells by monocyte-derived DCs maturated with heat shock-conditioned OEC lysates. Sorted CD4^+^ (a) and CD8^+^ (b) T cells were kept alone (LT alone) or cocultured for 14 days with autologous IL-4/GM-CSF-activated monocytes unstimulated (AM) or maturated for 24 hours with 100 *μ*g/mL of heat shock-conditioned lysates TRIMEL, Hey, MOVL1, or MOVL2 (TRIMEL-DC, Hey-DC, MOVL1-DC, and MOVL2-DC, respectively). The expression levels of CD25 and CXCR3 on CD4^+^ (a) or CD8^+^ (b) T cells were analyzed by flow cytometry and showed as mean fluorescence intensities (MFI). The results were obtained from multiple independent experiments. Statistics compare treatments to AM; ^∗^*p* < 0.05; ^∗∗^*p* < 0.01, and ^#^*p* < 0.001.

**Figure 6:**
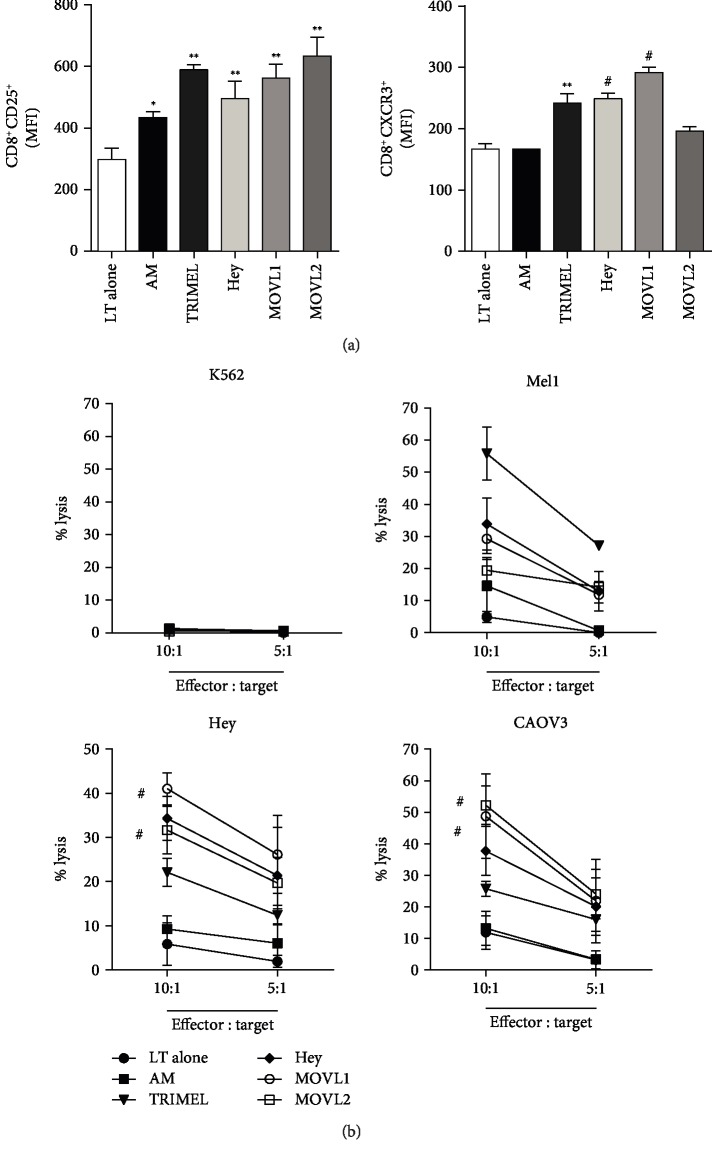
Monocyte-derived DCs maturated with heat shock-conditioned OEC lysates induce antitumor effector responses in autologous CD4^+^ and CD8^+^ T cells. Sorted CD4^+^ (a) and CD8^+^ (b) T cells were kept alone (LT alone) or cocultured for 14 days with autologous IL-4/GM-CSF-activated monocytes unstimulated (AM) or maturated for 24 hours with 100 *μ*g/mL of heat shock-conditioned lysates TRIMEL, Hey, MOVL1, or MOVL2 (TRIMEL-DC, Hey-DC, MOVL1-DC, and MOVL2-DC, respectively). After coculture with the different DCs, CD4^+^ (a) and CD8^+^ (b) T cells were sorted and challenged with different target tumor cells (Hey, CAOV3, Mel1, and K562). (a) IFN-*γ* secretion was evaluated by ELISPOT assays. (b) The lysis of the target cells was evaluated by ^51^Cr release assays. The results were obtained from multiple independent experiments. Statistics compare treatments to AM; ^∗∗^*p* < 0.01 and ^#^*p* < 0.001.

Given that heat shock-conditioned OECCL lysates potentially contain a large number of OEC-associated antigens that can produce a broad number of antigenic epitopes on DCs for priming T cell responses, we investigated whether CD4^+^ and CD8^+^ tumor-specific IFN-*γ*-secreting T cells were also being elicited *in vitro* by autologous HLA-A2^+^ MOVL1- or MOVL2-DCs. First, we observed that DCs pulsed with MOVL1 or MOVL2 lysates were able to activate autologous CD4^+^ and CD8^+^ T cells, measured by the expression level of CD25 or the chemokine receptor CXCR3 on T cells after 14 days of coculture (Figures 5(a) and 6(a)). Moreover, after coculture with autologous DCs, we evaluated the level of IFN-*γ* secretion by CD4^+^ T cells challenged with two HLA-A2^+^ OECCL present in the MOVL1 and MOVL2 lysates (Hey and CAOV3 cells) and HLA-A2^+^ melanoma cell line (Mel1) or with the leukemic cell line K562 (HLA-DR^−^) as negative controls.  Figure 5(b) shows that MOVL1- or MOVL2-DC-activated CD4^+^ T cells released significantly higher levels of IFN-*γ* after challenge with Hey or CAOV3 cells than CD4^+^ T cells unstimulated (LT alone) or cocultured with AM. Similarly, CD4^+^ T cells cocultured with TRIMEL-DCs were able to cross-recognize OEC cells in a discrete way, indicative of shared antigens between both kinds of tumor cells. Interestingly, CD4^+^ T cells activated with DC loaded with a heat shock-conditioned lysate made only from Hey cells (Hey-DC), despite having a similar level of cell activation (Figure 5(a)), released lower levels of IFN-*γ* after challenge with Hey or CAOV3 cells than CD4^+^ T cells cocultured with MOVL1/2-DCs (Figure 5(b)), suggesting a synergic or summative effect of using mixed OEC lysates. As expected, none of the CD4^+^ T cells released IFN-*γ* upon challenging with K562.

In a similar way, we studied the cytolytic activity of CD8^+^ T cells previously cocultured with autologous HLA-A2^+^ DCs pulsed with MOVL1 or MOVL2 lysates. CD8^+^ T cells were isolated after coculture by cell sorting and challenged with Hey, CAOV3, Mel1, and K562 cells.  Figure 6(b) shows that CD8^+^ T cells stimulated with MOVL1- or MOVL2-DCs were able to lyse Hey, CAOV3, and in a lesser extent Mel1 cells. Comparable to the results showed in Figure 6(a), CD8^+^ T cells activated with TRIMEL-DCs were able to cross-recognize OEC cells but in a lower degree. As expected, the K562 cells were not killed by any of the CD8^+^ T cells.

## 4. Discussion

Based on previous clinical findings, OEC has been recognized as a highly immunogenic tumor. Indeed, the presence of CD8^+^ tumor infiltrating lymphocytes is associated with an improved clinical outcome in late-stage ovarian cancer patients [37]. In addition, tumor-reactive T cells and antibodies can be detected in the blood and ascites of OEC patients with advanced disease. On the other hand, tumor-resident regulatory T cells are negatively correlated with prognosis in many cancer patients, including OEC [6]. In addition, in most OEC tumors, the tumor-reactive lymphocyte populations show impaired antitumor function *in vivo*, given that this particular tumor presents multiple mechanisms of immune evasion [38]. Nevertheless, immunogenic tumors as OEC can benefit from different immunotherapeutic interventions.

The use of therapeutic DC-based cancer vaccines as monotherapy or as complements to the ICB (such as anti-CTLA-4, anti-PD-1, or anti-PD-L1) may constitute a feasible possibility for improvement of clinical response rates in OEC patients, mainly due to their relative effectiveness in activating cell-mediated immune responses and their lack of severe side effects in cancer patients. The use of immunotherapeutic approaches, including DC vaccines, for OEC treatment has become an area of active investigation, and a number of clinical trials have been conducted or are still in development [39, 40].

The optimal delivery of tumor antigens is one of the most important factors for the success of DC-based anticancer vaccines. Superior clinical efficacy was consistently observed in cancer patients vaccinated with DCs pulsed with whole tumor lysates compared with DCs pulsed with defined tumor-associated peptides/proteins [41]. Autologous whole tumor antigens offer an unparalleled advantage as they allow DCs to process and present a broad range of tumor-associated antigens to stimulate strong, polyclonal, and long-term memory CD4^+^ and CD8^+^ T cell responses, potentially preventing tumor immune escape. Moreover, this strategy is suitable for all cancer patients regardless of their HLA haplotype. However, not all cancer patients have surgically removable tumors, and therefore, a useful and promising alternative is the preparation of allogeneic cancer cell lysates that have demonstrated to provide a standardized applicable source of tumor-specific antigens in patients with nonresectable tumors [28, 29].

Importantly, whole tumor lysates can be prepared in several ways, and the methods of inducing cell death, cell stress, or the chemical modification of proteins could impact the immunogenicity and efficacy of the therapy. Current immunogenic treatment modalities used for preconditioning tumor cell lysates include ultraviolet irradiation, oxidation-inducing modalities, and heat shock treatments [29, 32]. We previously showed that the heat shock treatment induces the release of well-established DAMPs, such as CRT and HMGB1, as well as putative DAMPs, such as haptoglobin. Melanoma and gallbladder cancer cells, and lysates generated from these cells, are immunogenic and clinically efficient [28–30, 42]. In the present study, we generated two heat shock-conditioned tumor lysates from mixtures of four different OEC cell lines (MOVL1: CAOV3, SKOV3, and Hey and MOVL2: CAOV3, SKOV3, and A2780), which showed important characteristics that suggest their potential as an antigen source for DC vaccines: (i) both lysate mixtures contain a broad panel of antigens relevant to OEC (Figure 1(a)); (ii) both lysate mixtures include different molecules that could act as DAMPs, such as released HMGB1 and translocated eCRT (Figure 1(b)); (iii) both lysate mixtures promote a rapid and efficient differentiation of monocytes to mature DC-like cells (Figure 2); and (iv) DCs generated with these lysates were able to induce the activation of both CD4^+^ and CD8^+^ T cells, which efficiently recognize and kill OEC tumor cells (Figures 3–6). Our results indicated that DCs loaded with heat shock-conditioned OEC lysates were able to induce the recognition of OEC cells by autologous CD4^+^ and CD8^+^ T cells (Figures 5 and 6). A major prerequisite for all immunotherapies that target MHC molecules (such as DC vaccines) is the persistent expression of these molecules on their target cancer cells. Recently, a high expression of HLA class I molecules on various ovarian tumors in both the RNA and protein levels without any evidence for HLA loss or downregulation [43] has been shown. The same report demonstrates that EOC strongly express also HLA-DR molecules, which correlate with the strong recognition of OEC cells by the DC-activated CD4^+^ T cells in our experiments (Figure 5(a)).

In conclusion, we propose that OEC heat shock-conditioned cell lysate-loaded DCs may be considered for future immunotherapy approaches alone or in combination with currently used immune checkpoint blocking therapies for ovarian cancer patients.

## Data Availability

The data used to support the discoveries of this study are available from the corresponding authors upon request to the email melopez@uchile.cl and fgonzalez@uchile.cl.

## Abbreviations

AM: Activated monocytes
APC: Antigen-presenting cells
CA-125: Cancer antigen 125
CCR7: C-C chemokine receptor type 7
CEA: Carcinoembryonic antigen
CRT: Calreticulin
CTLA-4: Cytotoxic T lymphocyte antigen-4
CXCR3: C-X-C motif chemokine receptor 3
DAMPs: Damage-associated molecular patterns
DCs: Dendritic cells
EDTA: Ethylenediamine tetra acetic acid
ELISA: Enzyme-linked immunosorbent assay
ELISPOT: Enzyme-linked immunosorbent spot
ErbB-2: Receptor tyrosine-protein kinase erbB-2
FBS: Fetal bovine serum
Hey-DCs: Dendritic cells matured with heat shock-conditioned Hey lysate
HLA: Human leucocyte antigen
HMGB1: High mobility group box-1
ICB: Immune checkpoint blockers
IFN-*γ*: Interferon gamma
LPS: Lipopolysaccharide
LPS-DCs: Dendritic cells matured with LPS
LT: Lymphocytes
mAb: Monoclonal antibody
MHC: Major histocompatibility complex
MOVL: Mixed ovarian carcinoma lysate
MOVL1/2-DCs: Dendritic cells matured with MOVL1 or MOVL2 lysates
MUC 1: Mucin-1
OEC: Ovarian epithelial carcinoma
OECCL: Ovarian epithelial carcinoma cell line
PBL: Peripheral blood lymphocyte
PBMC: Peripheral blood mononuclear cell
PBS: Phosphate-buffered saline
PD-1: Programmed death-1
PD-L1: Programmed death ligand-1
PMA: Phorbol myristate acetate
rhGM-CSF: Recombinant human granulocyte-macrophage colony-stimulating factor
rhIL: Recombinant human interleukin
SD: Standard deviation
Th1: Type 1 T helper cells
Th2: Type 2 T helper cells
Th17: Type 17 T helper cells
Treg: T regulatory cells
TRIMEL-DCs: Dendritic cells matured with TRIMEL lysate.
